# Intervention of pharmacist included in multidisciplinary team to reduce adverse drug event: a qualitative systematic review

**DOI:** 10.1186/s12913-023-09512-6

**Published:** 2023-08-30

**Authors:** Sarah Zaij, Kelly Pereira Maia, Géraldine Leguelinel-Blache, Clarisse Roux-Marson, Jean Marie Kinowski, Hélène Richard

**Affiliations:** 1 Department of Pharmacy, Nimes University Hospital, University of Montpellier, Nimes, France; 2 Desbrest Institute of Epidemiology and Public Health, Univ Montpellier, INSERM, Montpellier, France; 3 Department of Law and Health Economics, Faculty of Pharmacy, University of Montpellier, Montpellier, France

**Keywords:** Adverse drug event, Drug-related problems, Clinical pharmacist, Multidisciplinary team

## Abstract

**Background:**

Preventable harm in healthcare is a growing public health challenge. In addition to the economic costs of safety failures, adverse drug events (ADE) may lead to complication or even death. Multidisciplinary care team involving a pharmacist appears to be an adequate response to prevention of adverse drug event. This qualitative systematic review aims to identify and describe multidisciplinary planned team-based care involving at least one pharmacist to limit or prevent adverse drug events in the adult patients.

**Methods:**

To determine the type of interprofessional collaboration to prevent adverse drug event in which a pharmacist was involved, we conducted a qualitative systematic review of the literature of randomized controlled trials. Two independent reviewers screened trials in three databases: Medline, Web of Science, ScienceDirect. Prospective studies of at least three different health professionals’ interventions, one of whom was a pharmacist in the last five years were included. Two reviewers performed data extraction and quality appraisal independently. We used TIDieR checklist to appraise articles quality.

**Results:**

In total 803 citations were retrieved, 34 were analysed and 16 full-text articles were reviewed. Only 3 studies published an implementation evaluation. More than half of the interventions (62%) targeted elderly patients including 6 whom lived in nursing homes. Studies outcomes were heterogeneous, and we did not perform a statistical analysis of the impact of these interventions. Most teams are composed of a physician/pharmacist/nurse trio (94%; 100%; 88%). Half of the teams were composed of the primary care physician. Other professionals were included such as physical therapists (25%), social worker (19%), occupational therapists (12%), and community health educator (6%). Multidisciplinary medication review was the most common intervention and was generally structured in four steps: data collection and baseline assessment, appraisal report by health professionals, a multidisciplinary medication review meeting and a patient follow-up.

**Conclusions:**

The most common multidisciplinary intervention to prevent ADE in the adult population is the multidisciplinary drug review meeting at least the physician/pharmacist/nurse trio. Interventions target mostly elderly people in nursing homes, although complex chronic patients could benefit from this type of assessment.

**Trial registration:**

PROSPERO registration: CRD42022334685.

**Supplementary Information:**

The online version contains supplementary material available at 10.1186/s12913-023-09512-6.

## Background

Adverse drug events (ADE) represent a significant but mostly preventable clinical and economic burden [1]. ADE is defined as “any undesired event involving a drug that may or may not be preventable” [2]. In the scientific literature, this concept includes several terms: adverse drug reactions (ADR), drug-related problems (DRP) and medication errors (ME).

Between 1995 and 2000 in the United States, ADE related hospitalisation were estimated at between 1.8% and 7% [3]. In Europe, a systematic review estimated shows that the frequency of ADE resulting in hospitalizations 0.5% to 12.8% [4]. In France, the two national surveys ENEIS yielded similar results: almost half of the adverse events were associated with healthcare products (48% in 2004 and 58% in 2009). In total, drugs caused 1.5 to 2.1% of stays. In 2009, half of ADE were preventable (51.2%) and 54.5% resulted in hospitalisation [5, 6].

Many studies have shown that ADE causes are multifactorial. ADE are commonly related to the patient, its diseases, medication therapy or the care system. The most common risk factors are polypharmacy and lack of medication adherence [7–13]. In United States, 53% to 58% of ADR related hospitalization were due to medication errors [14].

Clinical pharmacy is one of the strategies to improve quality of medication therapy by optimizing therapeutic choices, dispensing, and administering medications to the patient. Pharmacists, both in ambulatory care and in hospital, is one of key strategies known to prevent DRP [15–17]. Their role is to advise healthcare professionals, educate patients, review the medications to ensure the quality of medicines provided to patients [18–20].

The Global patient safety action plan 2021–2030 published by the World Health Organization promotes a safety culture. It aims to eliminate avoidable harm in health care by optimizing the working environment. Multidisciplinary and interprofessional approaches are described as the new radical approaches needed to improve patient safety [21].

Ruiz-Ramos et al. systematic review and meta-analysis published in 2021 provides evidence that cooperation of pharmacist in a multidisciplinary team improves patient safety by reducing probability of readmission and patients’ quality of life while being cost-effective [22].

So far, there is no systematic review of the literature describing the whole process such as composition, health professional interactions, tools, and types of interventions of each stakeholder.

### Aim

The purpose of this qualitative systematic review was to identify and describe the planned care provided by a multidisciplinary team involving at least one pharmacist that aim to limit or prevent ADE in the adult patients.

## Methods

### Definitions

*Adverse drug event* (ADE) covers several keywords in in the scientific literature:

- *Adverse drug reaction* (ADR) defined as “a response to a drug which is noxious and unintended and which occurs at doses normally used in man for prophylaxis, diagnosis, or therapy of disease, or the modification of physiological function” [2].

Compared to ADR, an ADE does not prejudge a causal link with an exposure, in particular to a drug [23].

- *Drug-related problems* (DRP) are defined as “an event or circumstance involving drug therapy that actually or potentially interferes with desired health outcomes” [24].
- *Medication errors* (ME) are defined by The National Coordinating Council for Medication Error Reporting and Prevention as “any error in the process of ordering or delivering a medication regardless of whether an injury occurred or the potential for injury was present” [2].

A medication error or drug-related problem does not systematically lead to an ADE but increases the risk of occurrence. Less than 1% results in harm [2].

In the literature, the term *multidisciplinary team* is defined as “a group of professionals from two or more disciplines who work on the same project, independently or in parallel” [25].

The concept is also found as *multidisciplinary collaboration* which is a “process of problem-solving, shared responsibility for decision-making and the ability to carry out a care plan while working towards a common goal” [26, 27].

### Protocol and registration

The systematic review was conducted following PRISMA (Preferred Reporting Items for Systematic Reviews and Meta-Analyses) guidelines (Annexure 1). The systematic review protocol was registered with PROSPERO (International Prospective Register of Systematic Reviews) CRD42022334685 [28].

### Eligibility criteria

#### Types of studies

A recent systematic review published in 2021 analysing the impact of pharmaceutical care in multidisciplinary teams on health outcomes included randomized clinical trials published in 2000–2018 [22]. The purpose of our article was to update data. Moreover quality of publications on health interventions are often poor [29]. Indeed, we chose to select RCT that are high-quality articles which must respect reporting guidelines.

In the last five years the number of studies on multidisciplinary intervention including a pharmacist has increased significantly. Therefore, we chose to include all clinical randomized controlled trials published in the five past years in English (2017 to March 2022). Studies were included if the interventions described at least three health professionals in which a pharmacist was involved. We have chosen not to include multidisciplinary teams providing therapeutic education to focus on care pathways aimed at preventing or limiting ADE. Systematic reviews, reviews, protocols and theoretical articles were excluded.

#### Types of participants

Only studies involving adult patients were included, whether they were hospitalized or not.

### Information sources

We searched electronic databases including Medline, Web of Science and ScienceDirect using Medical Subject Headings and keywords related to: ADE, pharmacist intervention and multidisciplinary teams.

### Search

#### Study selection

Two authors (SZ, HR) performed screening of identified articles independently. Articles that met the inclusion criteria were included and reviewed. A third author (KPM) reviewed the search output and resolved any disagreements between reviewers.

#### Data collection process, quality, and risk of bias assessment

Selected citations were uploaded to a Zotero database. Study data were collected and managed using REDCap electronic data capture tools [30, 31]. Data of selected studies were analyzed using TIDIeR checklist (Template for Intervention Description and Replication) [29]. Each item on the assessment is worth one point. Studies with a score of less than 9 out of 12 points have been excluded from the systematic review.

#### Data items

Data were extracted using selected articles, their protocols and implementation studies, if published. The REDCap form created for data collection was used to extract information from selected articles. The form had several components: study caracteristics (population studied, inclusion and exclusion criteria, outcomes, relevant findings), interventions description and process (number and type of stakeholders, team coordination, communication with patients and follow-up) and finally the standardized tools used for intervention by health professionals. One form was filled out for each item selected. The results of the individual article was into an Excel spreadsheet. The results of all selected articles were summarized in three different Excel spreadsheets. These different tables are available in the results section. Missing data were specified in the tables.

## Results

### Study selection

**Fig. 1:**
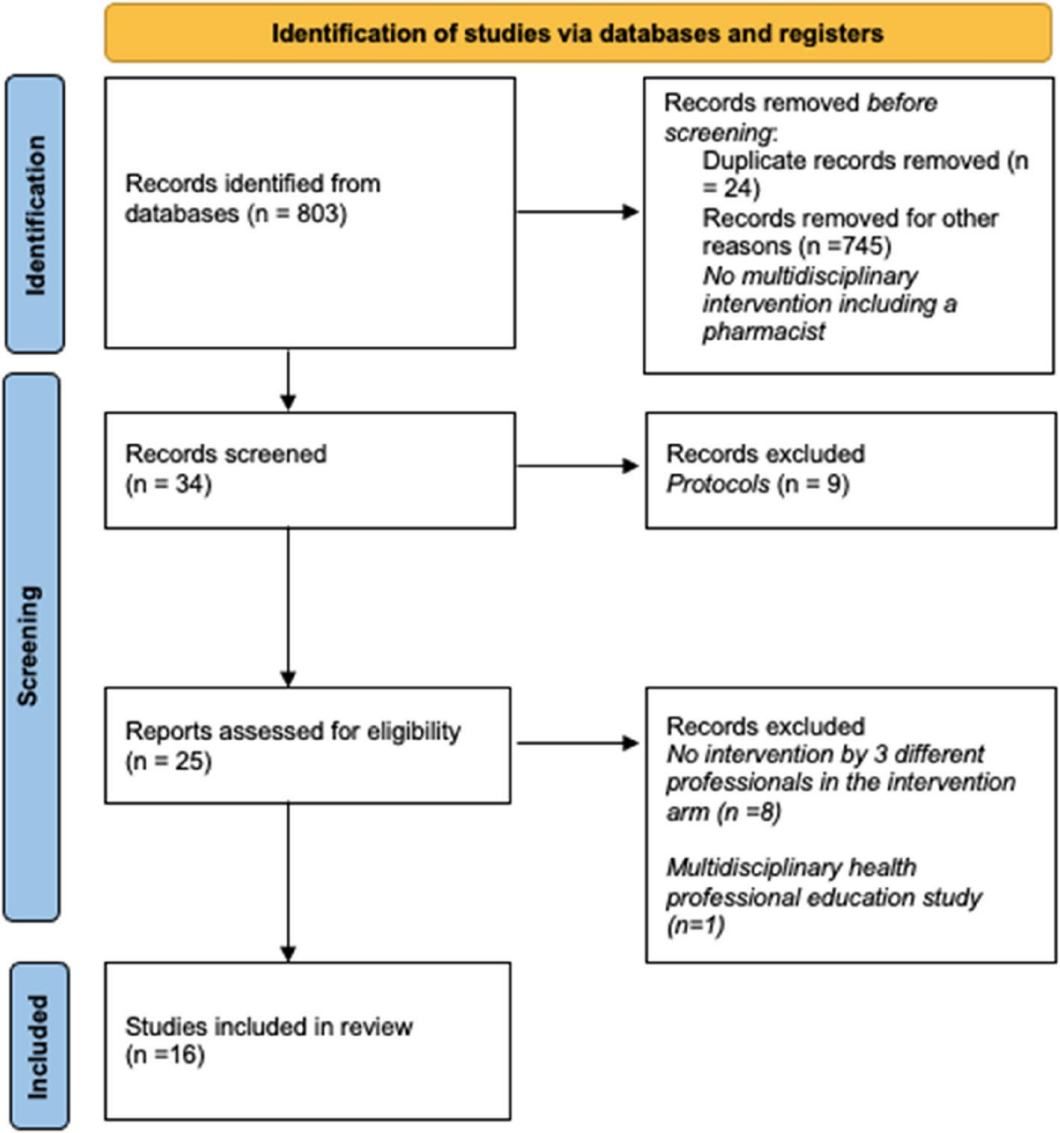
Article selection process

A total of 803 articles were retrieved from the 3 bibliographic sources. Of this selection, 737 articles were excluded because the study was not a randomized controlled trial, a pharmacist was not involved, or only 2 health professionals were involved. After removal of the 24 duplicates and 9 protocols, 33 articles were assessed for eligibility. In the end, 16 articles were selected and included in the systematic review [32–47]. Figure 1 shows the article selection process.

### Study characteristics

Table 1 summarizes the characteristics of the different multidisciplinary interventions included in the systematic review. Of the selected studies, 10 involved elderly patients, including 6 living in nursing homes [32, 33, 36, 37, 45, 46]. The interventions for adult patients that did not target the elderly focused on a specific chronic pathology (diabetes [42, 47], epilepsy [43], dementia [34], chronic pain treated with opioids [41] or pulmonary hypertension [38]).

**Table 1:** Studies characteristics

First author (year) (ref)	Country	Population	Sample size	Control group	Study outcomes	Results	Relevant findings
Cateau et al. (2021) [36]	Switzerland	Aged ≥ 65 years living in nursing home	*N* = 62	Usual care	1. Number and dose of PIM 2. QoL and safety outcome (mortality, falls, use of physical restraints)	1. No significantly decrease of number but significantly PIM dose decrease 2. No ADE were seen on mortality, hospitalizations, falls, and restraints use, but, in the intervention group, 3 patients experienced ADE that required the reintroduction of withdrawn treatments, and a decrease in QoL is possible	Intervention showed a potential benefit in the reduction of the doses of PIM used
Connolly et al. (2018) [45]	New-Zealand	Aged ≥ 65 years living in nursing home	*N* = 247	Usual care	Emergency presentations	25% reduction in ED presentations post-intervention	Intervention, targeted at selected conditions, decreases avoidable ED admissions of high-risk residents from selected facilities
DeBar et al. (2022) [41]	United-State	Aged ≥ 18 years	*N* = 850	Usual care	1.Pain impact (PEGS score) 2 Disability, satisfaction, opioid and benzodiazepine use	1. Reductions in PEGS score 2. Reduction in pain-related disability, higher satisfaction with primary care and pain services decrease in benzodiazepine use opioid use did not differ significantly	Intervention produced modest but sustained reductions in measures of pain and pain-related disability compared with usual care but did not reduce use of opioid medication
Desborough et al. (2020) [32]	United Kingdom	Aged ≥ 65 years living in nursing and residential home	*N* = 826	Usual care	1. Number of falls and PIM 2. Hospital admissions, deaths, economic evaluation	1. No significant differences for falls outcome, 20% decrease pims at 12 months 2. No significant difference ED admissions or deaths. Estimated cost was higher in the intervention group	Intervention improved medication appropriateness but failed to demonstrate improvements in clinical outcomes
Goldberg et al. (2020) [39]	United-State	Aged ≥ 65 years	*N* = 110	Usual care	Assess the feasibility and acceptability of the GAPCare (Geriatric Acute and Post-Acute Fall Prevention) intervention in the ED	98.2% received the pharmacy consult, 83,6% physical therapist consult ED length of stay was not increased in the intervention arm Pharmacy consult recommended by 100% of participants and 97.6% of clinicians. Physical therapist consult recommended by 95% of participants and 95.8% of clinicians	These findings support the feasibility and acceptability of the intervention in the ED
Kari et al. (2021) [35]	Finland	Aged ≥ 75 years	N = 277	Usual care	1. QoL 2. Physical performance, health resource use and cost, incremental cost effectiveness ratio	1. No difference in QoL 2. Physical performance: Unclear effect incremental cost effectiveness ratio -73 638 €/QALY	The cost-utility analysis showed that the intervention dominated usual care. However, it had no effect on QoL and the effect towards physical performance remained unclear
Levine et al. (2021) [44]	United-State	Aged ≥ 65 years living at home	N = 105	Usual care	Retrospective analysis of medication therapy problems related to cognition Classification: • Indication: underuse or overuse; • Effectiveness: ineffective agent or low dose; • Safety: ADE, unsafe medication (e.g., PIM), drug interaction, or high dose	Medication therapy problem (MTP) description: • 34% concern indication (79% underuse, 21% overuse) • 13% concern effectiveness • 52% concern safety: benzodiazepines and anticholinergics frequently implicated Recommendations: • 23% discontinuation • 19% dose reduction MTP involving cognition: among 79% patients	Intervention is effective to identify cognitively harmful medications, dementia treatment side effects, and untreated cognitive conditions
Li et al. (2021) [40]	United-State	Aged ≥ 65 years	N = 605	Usual care	1. ADE incidence 2. Hospitalizations (ED), chemotherapy dose	1. Significant 10.1% reduction incidence of grade 3 or higher chemotherapy-related toxic effects 2. No significant differences	Intervention significantly reduced grade 3 or higher chemotherapy-related toxic effects in older adults with cancer
Lu et al. (2021) [42]	China	18 years < aged < 75 years	N = 120	Usual care	1. Hb1Ac < 7% from baseline to 6 months 2. Changes in fasting blood glucose, daily medication cost, number of drug types taken daily and hypoglycemic events	1. Hb1Ac improved 2. No statistically significant decrease fasting blood glucose, costs. Significant decrease in number of medication types and hypoglycemic events	Intervention improved the rate of Hb1Ac for patients with type 2 diabetes mellitus
Possin et al. (2019) [34]	United-State	Aged > 45 years with dementia	N = 780	Usual care	1. Quality of life in Alzheimer’s disease 2. Hospitalization (ED) and use health services	1. Significant improvement of QoL 2. Decrease of ED visits, of caregiver depression and caregiver burden	Intervention mitigates the growing societal and economic burdens of dementia
Roustit et al. (2020) [38]	France	Patients with any type of pulmonary hypertension	N = 92	Usual care	1. Number of DRP (including ADE and ME) 2. Clinical worsening, adherence, quality of life and satisfaction with medications	1. No significant difference 2. No significant difference in time to clinical worsening, therapeutic adherence, satisfaction or QoL Collaborative care decreased costs of drug-related hospitalizations	Intervention improved the outcome of drug-related problems and reduced the costs of related hospitalization. However, we observed no efficacy on medication errors, clinical outcomes, or medication adherence
Siaw et al. (2018) [47]	Singapore	Aged ≥ 18 years	N = 411	Usual care	1. Hb1Ac, systolic blood pressure, low density lipoprotein and triglyceride 2.Evaluation emotional distress and treatment satisfaction, health service utilization rates and costs	1. Hb1Ac improvement of 0,8% 2. Improvements in PAID and DTSQ score, reduction in physician workload and average cost savings of $91,01 per patient	The positive clinical, humanistic, economic outcomes highlighted the value of multidisciplinary collaborative care for Asian diabetic patients, thereby supporting the effectiveness of this approach in managing chronic diseases
Strauven et al. (2019) [33]	Belgium	Aged ≥ 65 years living in nursing home	N = 1804	Usual care	1. PIM (success = at least 1 PIM solved) 2. Mortality and healthcare use and DRP	1. Significant effect in favor of the intervention 2. No significant difference between groups for most clinical outcomes	Intervention tested has successfully improved appropriateness of prescribing in nursing homes
Toivo et al. (2019) [37]	Finland	Aged ≥ 65 years living in nursing home	N = 129	Usual care	PIM	Intention-to-treat analysis shows no impact on the medication risks but the *per protocol* analysis shows tendency for effectiveness particularly in optimizing central nervous system medication use	Intervention indicated tendency for effectiveness when implemented as planned
Van Der Spek et al. (2018) [46]	Netherlands	Aged ≥ 65 years living in nursing home	N = 380	Usual care	Appropriateness of psychotropic drug prescription (APID score)	Significant improvement APID index sum score	The implementation of the intervention is effective
Zheng et al. (2019) [43]	China	Aged ≥ 18 years	N = 194	Usual care	Evaluation of depression, anxiety, medication adherence, QoL, number of seizures	Significantly reduced of patients with severe depression and anxiety Increased of patients with moderate-to-high adherence Increase QoL (QOLIE-31 score) Both groups: significant increase in patients with a low seizure frequency after 12-month	Intervention improved psychiatric comorbidities, medication adherence, and QoL

*ADE* adverse drug event, *APID score* Appropriateness of psychotropic drug prescription, *DRP* drug related problem, *ED* emergency department, *PEGS* Pain, Enjoyment, General Activity scale, *PIM* potentially inappropriate medication, *QoL* quality of life, *QALY* Quality-adjusted life year, *ME* medication error, *MTP* medication therapy problem

#### Quality appraisal

All randomised trials scored above 9 out of 12 on the TIDieR checklist appraisal. One of the items in TIDieR included implementation studies or evaluation studies of the intervention. Selected studies adopted different methods of implementation evaluation.

In two studies, adherence to protocol were provided by a committee or reviewed by phone calls [36, 46]. Two study organised management group meeting to set objectives, monitor progress and facilitate study delivery and evaluation [36, 46]. GAPCare study use descriptive statistics to assess key parameters of fidelity [39]. One study only evaluated number of completed intervention and deviation from protocol [45]. Three research team published a process evaluation article [48–50] from three of the selected interventions [33, 36, 41].

#### Results of individual studies

Outcomes of selected studies were heterogeneous. Regarding pharmaceutical criteria, six studies in the elderly or demented patients (in a nursing home or living at home) analysed inappropriate prescriptions rate using Beers criteria or STOPP/START tool (*n* = 6; 62%) [32, 33, 36, 37, 44, 46]. Studies in adult patients < 65 years assessed medication adherence [38, 43] (*n* = 2; 12%) or treatment satisfaction [41, 47] (*n* = 2; 12%). Roustit et al. study reported DRP or ME occurrence using NCC MERP Index for Categorizing Medication Errors tool [38] (*n* = 1; 6%). Studies in the elderly focused on cognitive and physical capacities, falls and mortality rate [32, 33, 35, 36]. Clinical endpoints assessed in studies also included disease balance mostly focusing on one disease [38, 41, 42, 47]. Five interventions evaluated cost-effectiveness of their model through health resource use and cost (e.g. hospitalizations type and rate, consultations, medication cost) [32, 35, 38, 42, 47]. Psychosocial criteria such as depression [34, 43], anxiety [43], emotional distress [47] and patient’s or caregivers’ quality of life [34–36, 38, 43, 47] were assessed.

Regarding medication treatments, one study showed a significant reduction by 20% in inappropriate prescriptions, one study showed improvement of the mean Appropriate Psychotropic drug use In Dementia index sum score in the intervention group, and another study showed a positive effect of the intervention of potentially inappropriate prescribing resolution [32, 33, 46]. Toivo et al. study intention-to-treat analysis [37] did not show an impact on the use of inappropriate medications (psychotropic, anticholinergic, and serotonergic), but the per-protocol analysis indicated a trend toward effectiveness. One study did not show a significant reduction in the number of chronic potentially inappropriate medication (PIMs) but was able to show a reduction in their dose after intervention by 28% [36]. GAIN intervention showed a 20% reduction in the incidence of grade 3 or greater chemotherapy-related toxicities [40]. Of the two studies that assessed patient medication adherence, Zheng et al. study showed improvement (moderate to high) to antiepileptic medication adherence [38, 43].

Two studies in diabetic patients, IMPACT intervention and Lu et al. study showed significant improvement in glycated hemoglobin measurement [42, 47]. Three studies that compared mortality rates between the two groups did not show significant improvement after intervention [32, 33, 36]. Two studies showed no impact of the intervention on the number of falls in the included elderly [32, 36]. Regarding hospitalizations, Connolly et al. study proved a 25% reduction in emergency presentation over 9 months after the intervention [45] and Possin et al. showed a significant reduction in emergency visits. Strauven et al., the median length of hospital stay was significantly longer in the control group. Two studies showed no impact on the number of hospitalizations after intervention [32, 40]. In terms of patient care pathways, two studies showed a significant reduction in emergency department presentations [45] and emergency department visits [34]. Roustit et al. did not show significant difference on clinical outcomes but demonstrated a cost decreased of drug-related hospitalizations [38]. In Kari et al., the incremental cost effectiveness ratio was valued at – 73 638€/ QALY [35].

Intervention decreased significantly caregiver depression and burden [34], reduced the number of patients with severe depression and anxiety [43] and improved patients evaluation of diabetes-related emotional distress [47]. Regarding patients’ or caregivers’ quality of life evaluation, two studies showed a significant improvement [34, 43].

Table 2 lists for each stage of the interventions, stakeholders, their missions, details of team coordination, communication with the patient modalities and patient’s follow-up. Intervention steps are sorted in chronological order. Information may be incomplete due to a lack of interventions description in articles.

**Table 2:** Interventions description and process

First author (year) (ref)	Stages of the intervention	Stakeholder	Team coordination	Communication with patient	Patient follow-up
Cateau et al. (2021) [36]	1. Deprescribing-focused medication review and adherence assessment, Quality of life evaluation	Pharmacist	Electronic and paper form	First visit in nursing home	Second visit 4 months after first one
	2. Creation of a treatment modification plan for each participant	Physician Pharmacist Nurse	Clinical staff (paper form)	Validation of the plan with the participant or her/his representative	
Connolly et al. (2018) [45]	1. Baseline assessment (identifying needs and facility care plan)	Gerontology nurse specialist facility senior nurse	3 one-hour meetings (1 per month, 6 residents considered per meeting)	*No information*	9 months before and 9 months after intervention
	2. Clinical coaching for nurses and caregivers	For nurses and caregivers			
	3. Multidisciplinary meetings: medication and care plan review	Study geriatrician Gerontology nurse Specialist Pharmacist General practitioner Senior nurse(s)			
DeBar et al. (2022) [41]	1. Comprehensive intake evaluation of pain (causes of pain exacerbation, impact functioning, depression, anxiety)	Nurse case manager or behavioral specialist	Biweekly telephone consultation with clinical investigators on the study	Face-to-face 2 sessions	12 weeks Individual phone coaching
	2. Medication review (potential alternatives to opioids or other adjustments of psychotropic medications)	Pharmacist		No information	
	3. Set physical activity goals, identify adaptations to be put in place for the adapted movement intervention)	Physical therapist		Face-to-face 1 session	
	4. Cognitive behavioral therapy-based pain coping skills training and adapted movement practice	Nurse case manager behavioral specialist	12 weekly group sessions with patients	12 weekly group sessions with healthcare professionals	
	5. PCP consultation and patient outreach	Nurse case manager Behavioral specialist Primary care physician	Face-to-face meeting	Additional phone calls	
Desborough et al. (2020) [32]	1. Medication review preparation (data extraction)	Pharmacist technician		*No information*	12 months
	2. Preparation pharmaceutical care plan	Clinical pharmacist			
	3. Multidisciplinary medication review	General practitioner Clinical pharmacist Pharmacist technician Care homes staff	Face-to-face 2 h meeting (15 residents considered per meeting) repeated after 6 months; Written details communicated to care home staff and community pharmacist		
Goldberg et al. (2020) [39]	1. Clinical assessment	ED clinician		Face-to-face	No follow-up
	2. Medication therapy management session: reconciliation, motivational interview, medication-related plan	Pharmacist	Written report to participant, ED team, and faxed to PCP	20 min face-to-face consultation	
	3. Fall risk assessment and plan	Physical therapist	Action plan in writing and in person to each participant and ED treatment team and faxed to PCP	20 min face-to-face consultation	
Kari et al. (2021) [35]	1. Self-care evaluation	Patient	Self-questionnaire		2 years
	2. Clinical medication review: reconciliation, medication review, recommandations	Pharmacist	Written report	Home visit	
	3. Health review	Nurse	Written report	Home visit	
	4. Interprofessional team meeting: discussion about DRP and health related issues, creation care plan	Physician Pharmacist Nurse	Face-to-face meeting	Nurse or pharmacist contact the patient to discuss the care plan	
Levine et al. (2021) [44]	1. Comprehensive clinical assessments and medication reconciliation (focused on cognitive impairment and depression)	Advanced pratice nurse	Weekly electronic communication, in-person meetings three times annually	4 home visits (2 h the 1rst and 1 h following) and 8 telephone contacts	12 months monthly call by the advanced practice nurse
	2. Medication assessment: identifying addressing, and helping resolve potential DRP	Clinical pharmacist		No direct contact with the patient	
	3. Conference to establish the comprehensive medication review: decision of recommendations to be forwarded to usual care providers via faxed letter	Geriatrician Psychiatrist advanced practice nurse			
	3. Problem Solving Therapy (PST) to patients with significant depressive symptoms	Licensed clinical social worker		6 weekly sessions face-to-face or by telephone	
	4. Modified protocol-driven Care for Persons with Dementia in their Environments (COPE) intervention	Occupational therapist		4 to 10 in-home visits	
	5. Protocol-driven Otago Exercise program	Physical therapist		4 in-home visits and 2 phone calls	
	6. Nutrition assessment	Dietician		In-home and telephone contact	
	7. Addressing social determinants of health	Community Health Educator		Letters and phone contact	
Li et al. (2021) [40]	1. Geriatric assessment: evaluation physical function, comorbidity, nutritional status, polypharmacy, social support, cognition, and psychological status	Patient Physician research team	*No information*	*No information*	Until chemotherapy completion or 6 months after initiation, whichever occurred first
	2. Multidisciplinary geriatric assessment review: intervention plan and appropriate referrals	Oncologist nurse practitioner social worker physical/occupation therapist nutritionist pharmacist	*No information*	*No information*	
	3. Additional support (patient education, care coordination, additional specialty referrals)	Nurse practitioner	*No information*	*No information*	
Lu et al. (2021) [42]	1. In-hospital pharmacy consultation, medication evaluation, and treatment	Physician Pharmacist Nurse		Face-to-face pharmacy consultations for each patient	1 week after discharge, once a week 1rst month, every 2 weeks months 2–3 and once a month until 6th month
	2. Out-of-hospital medication consultation, guidance, and adjustment of new treatment plans	Pharmacist physician	Web-based social media platform (WeChat)	Web-based social media platform (WeChat)	
Possin et al. (2019) [34]	1. Screening for problems, providing personalized support and standardized education	Care team navigator	Contact prescribing providers via secure facsimile	Phone calls	Monthly phone calls by the care team navigator and survey at baseline, 6 and 12 months
	2. Weekly case review and supervision	Care team navigator Advanced practice nurse social worker	Direct consultation with care team navigator	Direct consultation with patient and caregivers	
	3. Medication review at enrollment (recommendations send by the care team navigator to providers and caregivers)	Pharmacist	Discussion with advanced practice nurse	Consultation via telephone and secure messaging	
Roustit et al. (2020) [38]	1. Patient interview: medication review, identifying patient's needs, knowledge and skills, providing education	Pharmacist	Standardized report form	Face-to-face session at least once every 6 months	At baseline, 3, 6, 12 and 18 months
	2. Discussion of pharmacist's recommendations	Physician Pharmacist Nurse	Collaborative discussion		
Siaw et al. (2018) [47]	1. Patient diagnosis, assessment of diabetes severity, prescribing medications	Physician	*No information*	*No information*	Every 4 to 6 weeks for 6 months: face-to-face visits or phone calls by the clinical pharmacist
	2. Managing CAREPILLS^a^, optimizing medication using SIGN^b^ algorithm, furnishing prescription	Clinical pharmacist		Face-to-face session 20–30 min	
	3. Self-care counselling and basic foot and eye screenings	Diabetes nurse educator		Face-to-face session 20–30 min	
	4. Dietary counselling includes: carbohydrate counting, healthy food choices, weight management	Dietician		Face-to-face session 20–30 min	
Strauven et al. (2019) [33]	1. Blended training program (e-learning and workshops)	General practitioner (GP) Pharmacist nurse	Face-to-face workshops	No resident participation patient and family received feedback from the nurse or GP	15 months
	2. Local interdisciplinary meetings (discussion of the use of antidepressants and lipid-lowering drugs)		2 two-hours face-to-face meetings (baseline and month 3 or 4)		
	3. Interdisciplinary case conferences (medication review with web application)		20 min face-to-face meeting every 4 months		
Toivo et al. (2019) [37]	1. Risk assessment: medication reconciliation, DRP risk assessment, clinical tests	Practical nurse		Home visit	Baseline, 12 and 24 months
	2.Prescription review	Community pharmacist	Written report to physicians delivered by the nurse		
	3. Triage meetings: decision on actions for patients with clinically significant DRP	Coordinating pharmacist leading home care physician home care nurse	2 h face-to-face meeting		
	4. If a comprehensive medication review decided during triage meeting	Coordinating pharmacist	*No information*	Clinical patient interview and 3 months contacting practical nurses and/or patient	
	5. Case-conferences: collaborative medication review	Community pharmacist physician	Face-to-face meeting		
Van Der Spek et al. (2018) [46]	1. Education to medication review preparation	Physician Pharmacist Nurse			Baseline, 6, 12 and 18 months
	2. Conduct of multidisciplinary medication review at 0-, 6- and 12 months (focus psychotropic drugs use and neuropsychiatric symptoms)		Face-to-face meeting	Participants were not directly involved in the study	
	3. Evaluation meetings on the process at 6 and 12 months				
Zheng et al. (2019) [43]	1. Interview in epilepsy clinic (evaluation of epilepsy knowledge and self-management skills, and depression) ± score of BDI ≥ 16 or the score of BAI ≥ 37 ± score of MMAS8 < 6	Epileptologist ± psychiatrist ± pharmacist	*No information*	Face-to-face	4 weeks, 3 months intervals (for 12 months)
	2. Online consultation: educational information	Epilepsy specialist nurse		Web-based social media platform (WeChat)	
	3. Multidisciplinary education group	Epileptologist Psychiastrist epilepsy specialist nurse pharmacist Patient	Twice a year meeting in presence of patients		

^a^CAREPILLS: Closer monitoring, Adherence problem, Resistance to drug therapy, Empowerment in patient’s own therapy, Polypharmacy, Insulin titration, Lack in drug knowledge, Lack in drug administration techniques and Switching of drugs

^b^SIGN: Symptom-based Insulin adjustment for Glucose Normalization); PCP: primary care physician; ED: emergency department

Half of the intervention teams consisted of a tripartite team including a physician, a nurse and a pharmacist [32, 33, 35–38, 42, 46] (*n* = 8; 50%).

Physician are included in 15 of selected studies (94%). In one study, participants were referred by treating providers but the primary care physician did not provide care intervention [34]. In 50% of the studies, the team is composed of the primary care physician. Five studies involved specialist doctors: geriatricians [44, 45] (*n* = 2; 12%), psychiatrists [43, 44] (*n* = 2; 12%), emergency department clinicians [39] (*n* = 1; 6%), epileptologists [43] (*n* = 1; 6%). Generally, physicians were involved in the decision making of care plans during multidisciplinary meetings during which they retain possibility of refusing proposed modifications. In five studies patient received a clinical assessment from the physician [39, 40, 42, 43, 47].

Among the 16 studies, the level of training of the pharmacists was different. Four studies involved a clinical pharmacist [32, 38, 44, 45] (*n* = 4; 25%), three studies involved a community pharmacist [33, 35, 37] (*n* = 3; 19%), two studies had a hospital pharmacist as part of the team [39, 42] (*n* = 2; 12%) and seven studies did not specify the pharmacist's specialization [34, 40–42, 46] (*n* = 7; 44%). Three studies involved a “clinically trained” pharmacists [34, 36, 37] (*n* = 3; 19%). Medication reconciliation was performed by a pharmacist technician in one [32] and by a pharmacist in two studies [35, 39]. Pharmacists performed a medication assessment to identify, address and help to resolve potential DRP [44], proposed potential therapeutic alternatives, or adjustment medication [41]. They use standardised tools such as STOPP/START tool to review medication in the elderly patients [32, 33, 36, 46]. When seen in consultation with the patient (by telephone, home visit or face-to-face), they identified patient’s needs, knowledge, skills and provided motivation and education interview [35, 38, 39, 42, 43, 47]. They also helped them with new treatment plans [42], conceived pharmaceutical plan [32, 39, 40, 42, 45], provided prescription [47], and assessed medication adherence [36, 38].

Nurses were included in 14 of the 16 selected studies (88%). One study included “care home staff” without specifying its composition [32] and one study did not include a nurse [39]. Advanced practice nurse [34, 44] or specialist nurse (gerontology [45], epilepsy [43], diabetes educator [47]) took action in five studies (*n* = 5; 31%). They performed clinical assessment [35, 37, 40, 41, 44, 45, 47], medication reconciliation [37, 44], DRP risk assessment [37], patient education and care coordination [40, 41, 43, 47]. For example, in Levine’s study, advanced practice nurse performed an in-home battery of clinical assessments designed to gain deeper clinical understanding of dementia, depression, and delirium-related symptoms, as well as to guide clinical triggers for referral to other members of the team [44].

Other health professionals were involved: social workers [34, 40, 44] (*n* = 3; 19%), community health educator [44] (*n* = 1; 6%), physical therapists [39–41, 44] (*n* = 4; 25%), and occupational therapists [40, 44] (*n* = 2; 12%). In the Care Ecosytem intervention, community health worker connected the different health care professionals with each other and with the patient under nurse supervision [34]. In Possin et al. study, community health educator was a pivotal person who provided a link between the patient or caregiver and specialized health professionals [34].

**Fig. 2:**
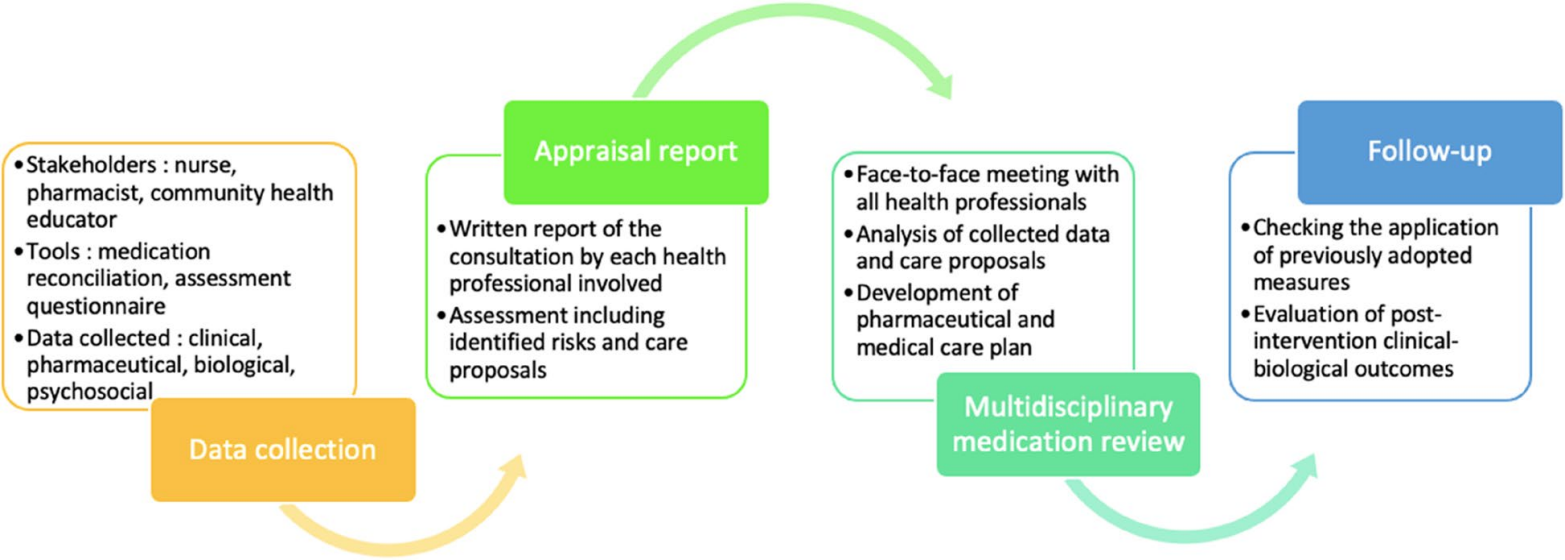
Interventions process

Interventions typically involved several steps (Fig. 2). The first is to collect clinical, biological, pharmaceutical, or social data relevant to the analysis. Most of the time, this research was done by the nurse, pharmacist, or a community health educator. Assessment questionnaires were filled in either by the health professional or by the patient himself. For example, in GAIN intervention, patient and health professionals complete an in-depth geriatric assessment. Patient portion included self-reported measures of (psychological state, social activity/support) and health care professional portion consisted of clinical assessment such as Karnofsky and Fulmer SPICES assessment [40].

Following data collection, the health professionals in their field of expertise prepare a detailed analysis report. Their analysis used standardized tools, the list of which is detailed (Table 3). These expert reports were either in the form of paper reports [32, 35–39] or electronic reports [36]. The purpose of this step was to develop a baseline assessment, to screen for problems, to identify the patient's needs, drug-related risks and to propose solutions.

**Table 3:** Standardized tools used in selected interventions

Evaluation	Tool used	Study
Pharmaceutical analysis	Systematic Tool to Reduce Inappropriate Prescribing (STRIP)	Van Der Spek et al. (2018) [46]
	STOPP/START tool	Desborough et al. (2020) [32]; Strauven et al. (2019) [33]; Cateau et al. (2021) [36]; Van Der Spek et al. (2018) [46]
	SFINX (electronic drug-drug interaction screening data base)	Toivo et al. (2019) [37]; Kari et al. (2021) [35]
	Appropriate Psychotropic drug use in dementia index	Van Der Spek et al. (2018) [46]
	Medication appropriateness Index (MAI)	Van Der Spek et al. (2018) [46]
	Beers criteria (2015)	Levine et al. (2021) [44]; Strauven et al. (2019) [33]; Toivo et al. (2019) [37]; Goldberg et al. (2020) [39]
Medication adherence	Morisky Medication Adherence Scale -8	Zheng et al. (2019) [43]; Kari et al. (2021) [35]; Roustit et al. (2020) [38]
Treatment satisfaction	Diabete Treatment Satisfaction Questionnaire (DTSQ)	Siaw et al. (2018) [47]
	Treatment Satisfaction with Medicines Questionnaires (SATMED-Q)	Roustit et al. (2020) [38]
DRP assessment risk	Drug Related Problem Risk Assessment TOOL (DRP-RAT)	Toivo et al. (2019) [37]
	Cancer and Aging Research Group (CARG) chemotherapy toxicity risk score,	Li et al. (2021) [40]
DRP classification	Basger et al. DRP classification	Strauven et al. (2019) [33]
	NCC MERP Index for Categorizing Medication Errors	Roustit et al. (2020) [38]
	PCNE DRP classification V6.2	Strauven et al. (2019) [33]
	SFPC Pharmacist Intervention classification	Roustit et al. (2020) [38]
	Cancer Institute Common Terminology Criteria for Adverse Events (version 4.0)	Li et al. (2021) [40]
DRP assessment of severity	Common Terminology Criteria for Adverse Events (CTCAE) v 5.0	Cateau et al. (2021) [36]
	IHI Global Trigger Tool for measuring Adverse Events	Roustit et al. (2020) [38]
	UKU Udvalg fo kliniske undersogleser side effect rating scale	Van Der Spek et al. (2018) [46]
DRP assessment of causality	CH E2A guidelines	Cateau et al. (2021) [36]
Quality of life	EQ 5D 5L	Cateau et al. (2021) [36]; DeBar et al. (2022) [41]
	QUALIDEM	Van Der Spek et al. (2018) [46]
	Short Form 36 Health Survey	Kari et al. (2021) [35]; Roustit et al. (2020) [38]
	Quality of life in Epilepsy 31	Zheng et al. (2019) [43]
	Quality of Life Alzheimer's Disease Scale	Levine et al. (2021) [44]; Possin et al. (2019) [34]
Depression, anxiety, emotional burden and sleep quality	Patient Health Questionnaire PHQ9 depression measure	Levine et al. (2021) [44]; Possin et al. (2019) [34]
	Beck Depression Inventory	Zheng et al. (2019) [43]
	Beck Anxiety Inventory	Zheng et al. (2019) [43]
	GRID Hamilton Rating Scale (GRID-HAMD)	Levine et al. (2021) [44]
	Center for Epidemiologic Studies—Depression scale	Levine et al. (2021) [44]
	Geriatric Depression Scale-15 (GDS 15)	Possin et al. (2019) [34]; Toivo et al. (2019) [37]
	Nijmegen Observer-Rated Depression scale (NORD)	Van Der Spek et al. (2018) [46]
	Minimum Data Set Depression Rating Scale (MDS-DRS)	Van Der Spek et al. (2018) [46]
	Zarit Burden Interview	Possin et al. (2019) [34]
	Caregiver Strain Index	Possin et al. (2019) [34]
	Problem Areas in Diabetes Questionnaire (PAID)	Siaw et al. (2018) [47]
	Pittsburgh Sleep Quality Index	Levine et al. (2021) [44]; Possin et al. (2019) [34]
Literacy assessment	Rapid Assessment of Adult Literacy	Possin et al. (2019) [34]
Social assessment	Medical Outcomes Study Social Support Survey Instrument	Li et al. (2021) [40]
	Medical Outcomes Study Social Activity Limitation Measure	Li et al. (2021) [40]
	Seeman and Berkman Social Ties	Li et al. (2021) [40]
	Revised Index of Social Engagement (RISE)	Van Der Spek et al. (2018) [46]
Daily activities Disability	WHO Disability Assessment Schedule	Levine et al. (2021) [44]
	Instrumental Activities of Daily Living (IADL)	Li et al. (2021) [40]
	Barthel Index for Activities of Daily Living (ADL)	Goldberg et al. (2020) [39]
	Minimum Data Set Resident Assessment Instrument (MDS-RAI)	Van Der Spek et al. (2018) [46]
	Roland Morris Questionnaire	DeBar et al. (2022) [41]
Pain assessment	PEG-3 Item Pain Scale	DeBar et al. (2022) [41]
	Pain assessment in Advance Dementia Tool	Possin et al. (2019) [34]
Clinical tools	Incontinence Impact Questionnaire	Levine et al. (2021) [44]
	Urogenital Distress Inventory	Levine et al. (2021) [44]
	Urinary Distress Inventory (UDI-6)	Toivo et al. (2019) [37]
	Mini Nutritional Assessment	Levine et al. (2021) [44]; Toivo et al. (2019) [37]
	Fulmer SPICES tool	Li et al. (2021) [40]
	Edmonton Symptom Assessment Scale (ESAS)	Possin et al. (2019) [34]
	CDC's STEADI Instrument	Goldberg et al. (2020) [39]
	Orthostatic hypotension test	Toivo et al. (2019) [37]
Cognitive assessment	Neuropsychiatric Inventory (NPI-Q) and Nursing Home version	Possin et al. (2019) [34]; Cateau et al. (2021) [36]; Van Der Spek et al. (2018) [46]
	Montreal Cognitive Assessment (moca)	Levine et al. (2021) [44]; Possin et al. (2019) [34]
	Six Item Screener	Goldberg et al. (2020) [39]
	Blessed Orientation Memory Concentration test (BOMC)	Li et al. (2021) [40]
	Karnosfsky Performance Status	Li et al. (2021) [40]
	Mini Mental State Examination (MMSE)	Toivo et al. (2019) [37]
	Severe Impairment Battery-8	Van Der Spek et al. (2018) [46]
	Cohen-Mansfield Agitation Inventory (CMAI)	Van Der Spek et al. (2018) [46]
	3D Confusion Assessment Method (CAM)	Levine et al. (2021) [44]
Functional assessment	Global Deterioration Scale (GDS)	Van Der Spek et al. (2018) [46]
	Short Performance Physical Battery (SPPB)	Kari et al. (2021) [35]
	Functional Assessment Staging Tool (FAST)	Possin et al. (2019) [34]
	Timed up and Go test	Levine et al. (2021) [44]; Li et al. (2021) [40]; Goldberg et al. (2020) [39]
	Five-times-sit-to-stand test	Toivo et al. (2019) [37]
Alcohol abuse detection	CAGE Substance Abuse Screening Tool	Levine et al. (2021) [44]
	Alcohol Use Disorder Identification Test version C (AUDIT-C)	Toivo et al. (2019) [37]

The third step was a multidisciplinary meeting bringing together all health professionals included. Ten studies performed a multidisciplinary medication review (*n* = 10; 62%) [32–37, 40, 44–46]. Together, health professionals reviewed patients’ medication plan considering their previous evaluation. Medication review helped them to identify inappropriate medications, reduce the number of medication errors, and increase frequency of monitoring. Multidisciplinary team decided on a care plan, a personalized medication plan and recommended a consultation with a health professional if necessary. For example, if pain was identified a supportive care/pain management referral was proposed [40]. In Levine's study, problem-solving therapy by a social worker in consultation or by teleconsultation was offered if significant depressive symptoms were detected during the clinical assessment at home by an advanced practice nurse [44]. After multidisciplinary meetings a feedback was given by the general practitioner or nurse in two studies to the patients or caregivers [33, 35].

Communication between health professionals took place mainly during interprofessional meetings. Meetings were held face-to-face, and duration and frequency were not always mentioned. The intervention lasted one hour [45] to two hours [32, 33, 37]. These meetings were weekly [45], quarterly [44], four-monthly [33], six-monthly [32] or annually [43]. Three studies did not report multidisciplinary meetings [39, 47, 48]. In GAPCare study, healthcare professionals shared information with each other in writing [39]. When the primary care team was not included in the multidisciplinary meeting, a report was sent to them [32, 37, 39]. In Lu et al. study, care team used WeChat app to treat and monitor patient’s medications [42].

Communication with patient took place mainly through consultations in 11 studies. Of these, six were performed by the nurse [34, 35, 37, 41, 47, 48], seven by the pharmacist [35, 36, 38, 39, 42, 43, 47]. In Levine's study, pharmacist had no direct contact with the patient. In two studies, patients were contacted by the pharmacist by phone or via an app [34, 42]. Patients could use WeChat for medication consultation, and pharmacists could reply. It was also used to follow the patients regularly to collect information on the treatment effects or adverse events. For 3 studies no information was found on the communication with the patient [32, 40, 45].

A follow-up in consultation or by phone was organized to evaluate the implementation of the decided modifications and to follow the clinical evolution of the patient. Only one study did not follow up the included patients [39]. Patients were followed up between 12 and 24 months. This follow-up was organized in person or by phone with the nurse [44], pharmacist [47] or community health educator [34].

Table 3 classifies tools used according to assessed outcome. Thirteen articles used standardised tools to assess patients' treatment-related aspects (satisfaction, compliance, risk of DRP, severity and causality, drug interaction, appropriateness), clinical aspects (disability, physical abilities) quality of life, social skills (literacy, social appraisal) and psychological aspects (depression, anxiety, emotional burden, sleep quality, pain) [33–41, 43, 44, 46, 47]. To analyse prescriptions, the pharmacist could use different tools such as STOPP/START criteria for potentially inappropriate prescribing for older people [51], drug-drug interaction database SFINX [52], Medication Appropriateness Index [53], Systematic Tool to Reduce Inappropriate Prescribing STRIP [54] or Beers criteria [55]. To assess DRP risk, pharmacist or nurse used Drug Related Problem Risk Assessment tool [56] or Cancer and Aging Research Group chemotherapy toxicity risk score [57]. The Morisky score was the only medication adherence assessment score used in the studies [35, 38, 43]. Two studies assessed patients' satisfaction with treatment [38, 47]. Pharmacists used different DRP classifications including PCNE DRP classification V6.2 and NCC MERP Index for Categorizing Medication Errors [33, 38, 40]. To assess quality of life, some tools were filled in either by the patient himself or other professionals. We found thirteen different tools used to assess depression, anxiety, burden, or sleep disorder including Patient Health Questionnaire PHQ9 depression measure [58] and GRID Hamilton Rating Scale [59].

## Discussion

In this qualitative systematic review, 16 studies that established a multidisciplinary intervention involving a pharmacist in adult patients were identified. Multidisciplinary medication review meetings were the most common clinical pathway involving a pharmacist found in this study and could represent a gold standard intervention to prevent ADE. Medication review is a structured evaluation of a patient medicines aiming to optimize prescriptions and improving health outcomes. In this care practice, decisions are shared between professionals regarding a goal to be achieved. A systematic review proved that medication review and reconciliation with cooperation between pharmacist and general practitioner decreased significantly number of DRP, improved prescribing of medication, improved quality of life scores, improved medication appropriateness index scores, increased compliance and patient knowledge, and improved clinical values [60]. These results were also confirmed by a recently published literature review which found that multidisciplinary intervention including pharmaceutical services (medication reconciliation and review) significantly increased patients’ quality of life (OR 0,58, 95% CI 0,47–0,69) and reduced the probability of readmission by 32% [22].

Multidisciplinary medication review was generally structured in four steps: baseline assessment including clinical, pharmaceutical, biological, and psychosocial relevant data collection, preparation of a detailed analysis report by health professionals in their field of expertise, a multidisciplinary medication review meeting and a patient follow-up. There was no list of standardised medication review activities published but the different stages of the process we found were consistent with activities found in an international policy review published in 2020 that compared medication review of 6 countries [61]. In this review, comprehensive patient interview and interprofessional collaboration, found in our process, were judged clinically important.

The presence of a leader who promoted the implementation of the intervention interdisciplinary, face-to-face approaches and positive attitude by general practitioners were acknowledged as a facilitator [33]. Intervention required healthcare professionals involvement and active role, a reinforced interprofessional collaboration with information sharing [35].

Our study showed that most teams were composed of a physician/pharmacist/nurse trio (94%; 100%; 88%). This results concords with a recent systematic reviewed that selected 29 studies from 2000 to 2018 in which multidisciplinary teams included pharmacists (*n* = 29;100%), physicians (*n* = 27; 93%) nurses (*n* = 15; 52%). The composition of the team is changing, and the pharmacist/doctor duo that used to be most common now includes a nurse almost systematically. Moreover, in this systematic review other professionals were included such as psychologists (*n* = 3; 10%) and occupational therapists (*n* = 2; 7%). Our results showed the presence of social workers (*n* = 3; 19%) and community health educator (*n* = 1; 6%), physical therapists (*n* = 4; 25%), occupational therapists (*n* = 2; 12%). Highly specialised teams associating tripartite team to social worker and/or specialist professionals or/and physiotherapists are beginning to emerge.

Pharmacists mostly performed deprescription intervention [36] and medication review based on clinical practices guidelines to prevent and limit two of the main causes of ADE in older people: polypharmacy and inappropriate prescribing [32, 33, 36, 37, 39, 44, 46]. Indeed, elderly people (> 65 years) were targeted in more than 60% (*n* = 10) of the interventions. Most of them lived in nursing homes (*n* = 6). They constitute a specific population because of the frequent occurrence of polypathologies, existence of physical, psychological, or socio-economic fragility and a risk of loss of autonomy and dependence [51–54]. However, polypathology are also common in non-elderly patient with a frequency of patients aged 40–64 reported an average of 3.4 pathologies per person [62]. In our study, interventions for non-elderly people with chronic diseases, pharmacist and team interventions were focused on the disease (chronic pain, epilepsy, diabetes) and patient did not benefit from a global assessment as seen in elderly interventions. Patients eligible for this type of intervention should be selected based on the complexity of their needs and not only on their age. Moreover, only two studies assessed medication adherence [38, 43]. Yet, medication non-adherence is a main cause of lack of optimal clinical benefit [63, 64]. It can lead to medical and psychosocial complications of the disease, reduce patients' quality of life, waste healthcare resources [65] and potentially lead to ADE [66]. This reinforces the pharmacist’s key role in the patient’s overall assessment to prevent or limit ADE.

Involvement of the nurse in patient's care and in multidisciplinary decision-making is essential. In selected studies, their proximity to patients allowed them to speak on their behalf in multidisciplinary meetings. Nurses may be part of the patient's primary care team or may intervene during a consultation to provide care coordination, education, and in-home clinical assessment. Indeed, due to their training, nurses can detect, report, and monitor ADE emergence. In France, a recent decree specified that advanced practice nurses training should include polypharmacy identification and preparation of assessment for a multiprofessional consultation on medication reconciliation and ADE risk assessment, medication side effect identification, adherence medication monitoring, and signs of iatrogenic pathology identification throughout patient's care pathway [67].

Primary care physician’s role is central. Indeed, they have a global vision of the state of health of their patients and are the pivotal person of the medication iatrogenic risk prevention in ambulatory care. Optimising medication prescribing by reassessing the benefit/risk ratio, carrying out regular clinical and biological monitoring and prioritising pathologies in consultation with the patient helps to limit risk of ADE. Physicians reluctant attitudes and weak engagement were evaluated as the main contributing factors for intervention not being implemented [37]. Building trust among healthcare professionals and between the professionals is essential for effective collaboration [35]. Six interventions of selected studies included a specialist physician but only geriatrics and psychiatrist in Levine study [44] were included in a multidisciplinary consultation meeting. Epileptologist, and emergency department clinician only performed consultation with patient and referrals to other healthcare professionals. Inclusion of specialist physicians such as nephrologists, geriatricians, cardiologists could be considered during multidisciplinary medication reviews. Indeed, their skills are essential for making decisions that give patients the best possible care according to the state of science especially for multimorbid and complex patients.

A systematic review identified five main ADE risk factor categories: patient-related, pathology-related, drug treatment-related, care pathway-related and finally factors related to the patient's genetics [68]. Other risk factors (e.g. polypharmacy, polypathology) were identified such as psychosocial factors or complexity of patient’s care path. Thus, it is legitimate to include in the team other health professionals (social worker, psychologist, physical therapist) who are better able to assess the patient and propose appropriate solutions when an at-risk situation is detected. Three studies included social worker in the multidisciplinary team. A combined pharmacist and a social worker-led program to address psychosocial factors demonstrated a significant reduction in 30-day, all-cause readmission rates to the same hospital [69]. The inclusion of social workers in the patient's care pathway allows detecting depression, anxiety, or social withdrawal. Moreover, psychosocial support may also decipher medication non-adherence meaning, detect suffering, levers, obstacles to medication adherence, and assess patient's resources attitudes, perception, and beliefs assessment.

To appraise study quality, we selected randomized controlled trials and we used TIDieR checklist [29]. This checklist was developed to improve the completeness of reporting, and the replicability of interventions. It allowed us to highlight the lack of description of the implementation evaluation (acceptability, appropriateness, feasibility, fidelity) in selected studies. Only three studies published a process, an implementation evaluation or a qualitative study about their intervention [49, 50, 70], this studies have generally reported good results. However, some limitations have been highlighted. Concerns about feasibility, mainly due to time and resource constraints [33, 35–37, 40, 44, 47] were raised. For example, pharmacist did not participate in home-based care team in Levine’s study owing to limited time and resources, which represented a potential weakness [44]. To limit time constraint, GAIN intervention developers will assess the feasibility of implementing geriatric assessment driven interventions via telehealth in community practice [40]. In the GAPCare intervention board-certified resident pharmacists were trained to supplement pharmacist’s activities [39]. In Kari et al. study, it seemed important to better target patients who were most likely to benefit from these time-consuming interventions [35]. It would be beneficial to encourage health professionals working in multidisciplinary teams to publish their implementation studies to inform health intervention research, enable replication of the intervention and increase the potential impact of research on health.

Our descriptive analysis did not lead us to perform quantitative analysis however it would be interesting to extend the research question with a quantitative analysis with meta-analysis to evaluate the impact of multidisciplinary management to a conventional management.

### Limitations

This qualitative systematic review only included randomized controlled trials for quality reasons. Therefore, we did not review multidisciplinary interventions assessed with other methods. The inclusion of grey literature could have had a real benefit for our research question but it also requires a huge amount of time and resource to search. We have chosen to select only 3 reliable sources and not to include the grey literature, however it would be interesting to take the time to compare the results we were able to obtain with the results that the grey literature would have given us. We did not perform a quantitative analysis due to heterogeneity in outcome, intervention methods, participants’ demographics and settings of the included studies. However, we attempted to examine included studies using the standardised TIDieR checklist. Another potential limitation is the language selection; we only included articles published in English. It would be interesting to extend the search to articles written in other languages. Quality of this synthesis also depended on available data in intervention description; some information was not found and may limit our findings.

## Conclusions

This article is the first systematic review selecting randomised clinical trial and their implementation studies to analyse the process of multidisciplinary care including a pharmacist. In the context of growing complex care, multidisciplinary medication review meetings appear to be the common structure to ensure the effectiveness and safety of care. Essential to the diagnosis and management of patients, these meetings gather all the health professionals essential during four stages (data collection, appraisal report, multidisciplinary meeting, and follow-up) to establish a coordinated care plan. The comprehensive assessment of complex chronic patients by the tripartite team of physician, pharmacist and nurse should be completed with other professionals’ skills to consider all the ADE risk factors described in the literature.

### Supplementary Information

**Additional file 1.**

## Data Availability

All data generated or analysed during this study are included in this published article.

## Abbreviations

ADE: Adverse Drug Event
ADR: Adverse Drug Reactions
DRP: Drug-Related Problems
ENEIS: Enquête Nationale sur les Evénements Indésirables liés aux Soins (French national survey on adverse events in healthcare)
ME: Medication errors
NCC MERP Index for Categorizing Medication Errors: National coordinationg council for medication error reporting and prevention Index for Categorizing Medication Errors
PCNE DRP classification: Pharmaceutical Care Network Europe Drug Related Problem classification
PHQ9: Patient Health Questionnaire 9
PRISMA: Preferred Reporting Items for Systematic Reviews and Meta-Analyses
QALY: Quality-adjusted life year
REDcap: Research Electronic Data Capture
STOPP/START: Screening Tool of Older People's Prescriptions/ Screening Tool to Alert to Right Treatment
STRIP: Systematic Tool to Reduce Inappropriate Prescribing
TIDIeR: Template for Intervention Description and Replication
